# Bibliometric study on the knowledge graph of immunotherapy for head and neck cancer

**DOI:** 10.3389/fonc.2023.942777

**Published:** 2023-02-03

**Authors:** Ji Wang, Zhengpeng Gong, Ming Yu

**Affiliations:** ^1^ Department of Clinical Medicine, Guizhou Medical University, Guiyang, China; ^2^ Key Laboratory of Endemic and Ethnic Diseases, Ministry of Education & Key Laboratory of Medical Molecular Biology of Guizhou Province, Guizhou Medical University, Guiyang, China; ^3^ Department of Otorhinolaryngology Head and Neck Surgery, Affiliated Hospital of Guizhou Medical University, Guiyang, China

**Keywords:** head and neck squamous cell carcinoma, head and neck cancer, Immunotherapy, bibliometric study, VOSviewer, CiteSpace

## Abstract

**Background:**

Head and neck squamous cell carcinoma (HNSCC) is a common malignant tumor with a significant mortality rate, especially in patients at locally advanced stage, or with recurrence and metastasis. Immunotherapy has shown remarkable breakthrough in the treatment of locally advanced cancer, recurrence and metastasis in recent years. During this time, a large number of HNSCC immunotherapy studies have been published. However, few studies employed bibliometric analysis. This work analyzes HNSCC immunotherapy trends and hotspots using bibliometric analysis to get better understanding of the current state and future direction of HNSCC immunotherapy.

**Methods:**

Relevant articles and publications about immunotherapy of HNSCC were extracted from Web of Science Core Collection (WoSCC). Bibliometrics was used to study these publications in terms of countries/regions, institutions, authors (cited authors), journals (cited journals), references, and keywords, so as to identify research hotspots and to predict future research trends in this field.

**Results:**

A total of 1377 English articles published between 2000 and 2022 were collected. It is found that the number of articles increases rapidly from 2016. The United States has the largest number of publications (n=538), followed by China (n=407) and Germany (n=175). The institute with the highest published papers is the University of Pittsburgh (n=67). In terms of author, Robert L Ferris ranks first among the top ten cited authors. Oral Oncology (impact factor (IF) (2021) = 5.972) is the most prolific academic journal in immunotherapy of HNSCC. According to the reference cluster analysis, the research hot topic has shifted from basic research on immunotherapy of head and neck cancer to the study of prognosis. Keywords analysis also reveals that the study of patients’ prognoses is at the core of immunotherapy for HNSCC.

**Conclusion:**

Currently, head and neck cancer research focus primarily on prognostic significance, cancer treatment, and poor prognosis. However, the researches on immunotherapy for head and neck malignancies is the growing trend in near future. Notably, United States has made significant contributions to this field.

## Introduction

Head and neck cancer affects around 830,000 people worldwide each year, which results an estimated death of 430,000 people (1, 2). A combination of surgery, radiation, and chemotherapy can successfully treat many patients with locally advanced HNSCC. Nevertheless, some individuals develop recurrent/metastatic (R/M) HNSCC and are resistant to the above treatments. Only 40–50% of people with carcinogen-related head and neck squamous cell carcinoma (HNSCC) could survive 5 years after diagnosis despite the aggressive use of many treatment modalities, including surgery, RT, and chemotherapy (3). Patients who develop recurrence or distant metastases have few therapeutic options and a poor prognosis. Their median survival time after diagnosis is less than one year (3, 4). Immunosuppression exists within HNSCC tissues. Fewer lymphocytes, natural killer (NK) cells, and particular antigens are present than those in normal tissues. This sort of cancer is hence known as a “cold tumor”. Multiple methods allow HNSCC to avoid immune monitoring. Immune checkpoint inhibitors (ICIs) target the T cell regulatory pathway to enhance the efficacy of anti-tumor immunity, which represents a significant clinical advance and a novel approach to cancer treatment. In addition to surgery, radiation, and chemotherapy, targeted immunological checkpoints have emerged as a viable treatment option for HNSCC (5, 6). Immunotherapies have increased the survival rate of HNSCC patients, although the long-term prognosis of individuals with recurrent or metastatic HNSCC remains dismal (7, 8). To some extent, this is due to the expression of T-cell–suppressing immune-checkpoint receptor programmed death 1 programmed death ligands (PD-L1 and PD-L2) (PD-1) (9, 10). The immune system can evade anticancer immunotherapy due to changes in immune surveillance and the tumor microenvironment(TME) (3). T lymphocytes, natural killer cells, and antigen presentation function may be impaired in HNSCC patients due to upregulation of PD-1 and other immunological checkpoints receptor (ICR) molecules (11–14). Even though only a tiny fraction of patients reacts to immunotherapy when it is used alone as a treatment, the responses that are noticed are frequently long-lasting and profound. The extremely encouraging outcomes of treatment with these medicines have contributed to a surge in interest in the immune system and how it might be utilized in the fight against cancer.

Bibliometrics analysis employs the number of citations as an index to determine the quality of research, which is a useful tool for statistical and qualitative evaluation of the trend of research outcomes (15). It objectively evaluates the contributions of academic groups and individuals through a comprehensive analysis of the countries, institutions, authors, journals, and citations of selected articles, thereby providing a means of comprehending trends in particular fields and ranking academic groups and individuals (16–18). In addition, the modifications of frequently used keywords and research buzzwords in the collected articles are examined to give evidence in support of the predicted future trend (19, 20). At present, the bibliometric analysis of literature is mainly carried out using CiteSpace and VOSviewer. Many researchers have used this strategy to evaluate their respective fields of study (19, 21). There has been no specific bibliometric research on the knowledge graph of HNSCC immunotherapy to date. This study’s objective is to analyze the literature on HNSCC immunotherapy from 2000 to 2022 to assess the current state of the field and identify its future research directions.

## Materials and methods

### Data sources and search strategies

For bibliometric analysis, the Web of Science Core Collection(WoSCC) database from Clarivate Analytics was used as the standard (22). Using the WoSCC database, we looked through all of the published research in English on HNSCC immunotherapy between 2000 and 2022 (Retrieval time:2000.01.01; Retrieval deadline: 2022.12.31) and only included original articles. Nasopharyngeal cancer is not explored because it has distinct epidemiology, pathology, natural history, and treatments that fall outside the focus of this research (23). We performed a Boolean search on two lists with the operator “and” The first list included the following search structure: TS = (“head and neck carcinoma” or “head and neck cancer” or “head and neck Squamous cell carcinoma” or “HNSCC” or “Oral squamous cell carcinoma” or”OSCC” or “laryngeal carcinoma”or “ hypopharyngeal carcinoma”). The second list included the following search structure: TS= (“immunotherapy” or “immunotherapeutic” or “Immunotherapies”). All data downloads and literature searches were completed on 15^th^ Jan 2023, to avoid potential bias from frequently updated databases.Two authors independently searched the WoSCC database for relevant literature and downloaded in TXT format (title, keyword, author information, abstract, reference, etc.). Any disagreements were resolved through consultation or by enlisting assistance coming from external experts to reach a consensus.

### Bibliometrics and visualization analysis

**Figure 1:**
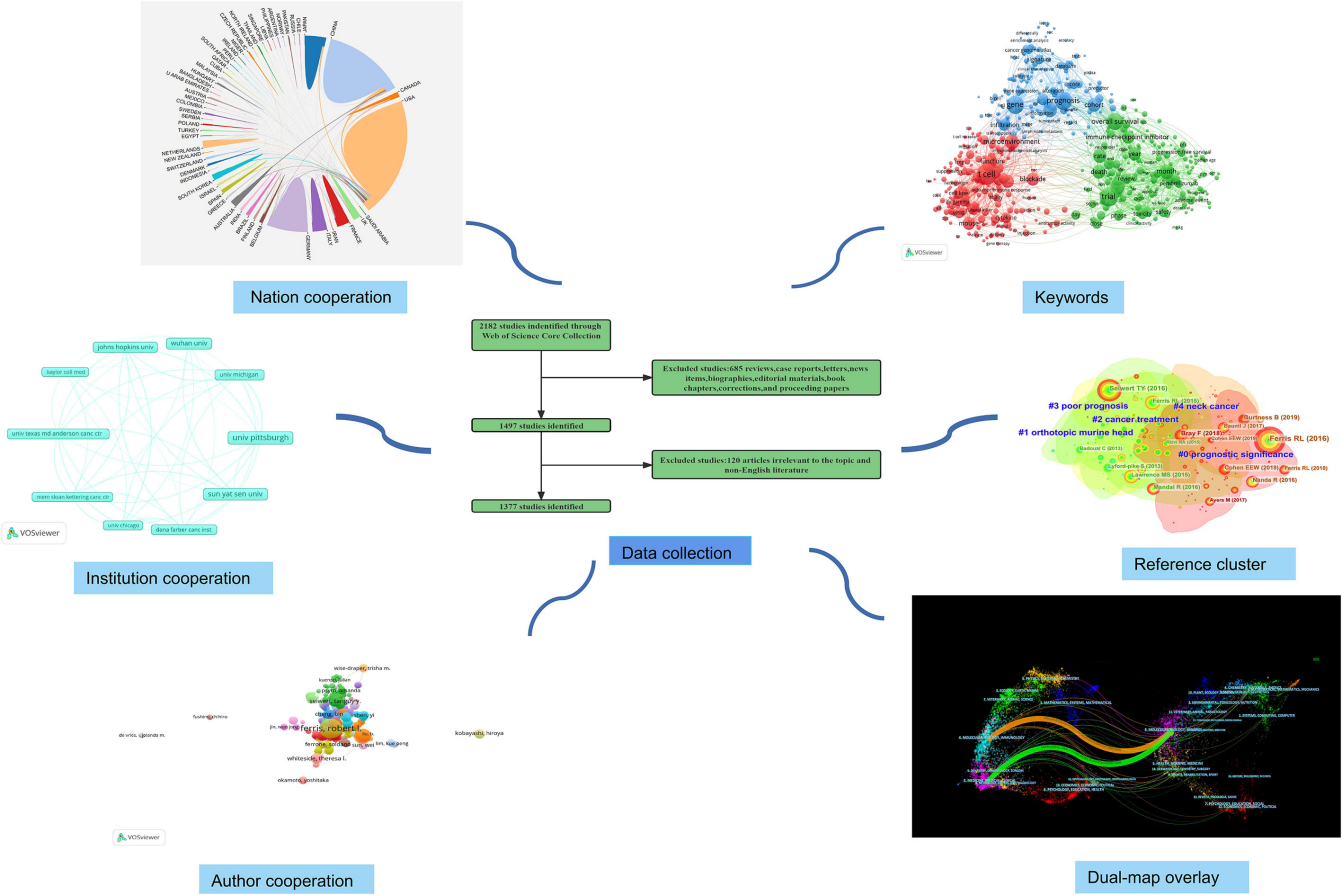
In order to forecast future research hotspots in this area, we searched the Web of Science Core Collection (WOSCC) for articles on the immunotherapy of HNSCC and carried out a visualization analysis from the perspectives of countries, institutions, authors, journals, references, and keywords.

All valid data were converted to text and then imported into the analysis software. Microsoft Word 2019 was used to create the annual publication and descriptive statistics on ranks and frequency. CiteSpace (6.1.R6), VOSviewer (1.6.18), and the bibliometric online analysis platform (http://bibliometric.com/) were used to identify countries, institutions, authors, journals, references, keywords, and network characteristics of keyword bursts, and to present the results visually (**Figure 1**). These programs were used for bibliometrics and visual analysis, respectively, to give essential information on the current immunotherapy understanding for HNSCC. When mapping visual knowledge graph, we follow the main procedure steps of CiteSpace, including time slicing, thresholding, modeling, pruning, merging, and mapping. The core concepts of CiteSpace include burst detection, intermediary centrality, and heterogeneous networks, which help to visualize research status, hotspots, and frontiers promptly. Nodes in different maps represent institutions or references. The node’s size indicates the frequency of occurrence or reference, and the node’s color indicates the year of occurrence or reference. In addition, nodes with red trimming represent high mediation centrality, which is usually identified as a hot or turning point in the field (24, 25). Use the VOSviewer to explore collaborative networks between authors, co-authors, and keywords, and the size of nodes is determined by their frequency of co-occurrence in titles and abstracts (20).

## Results

### Data collection

**Figure 2:**
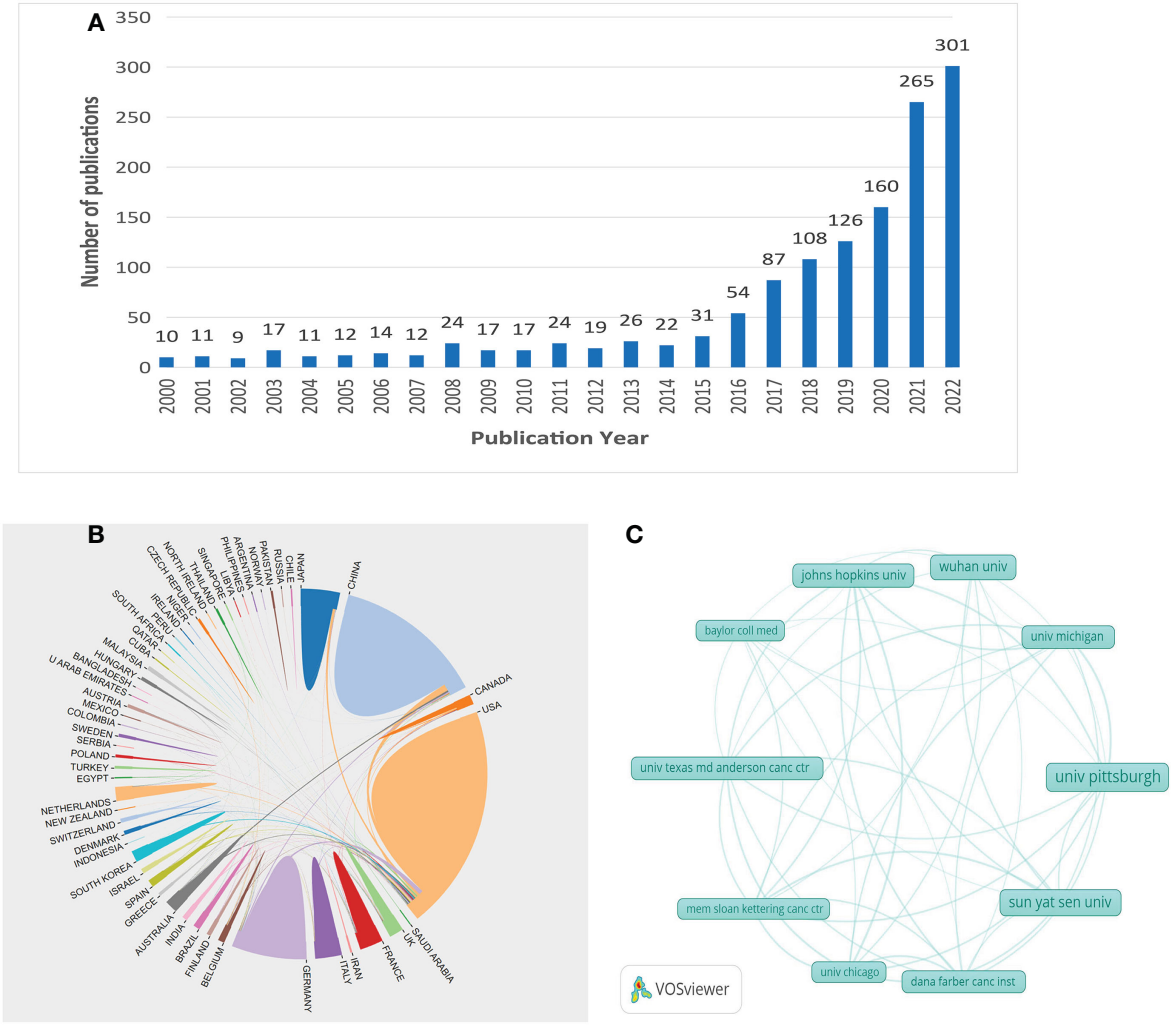
From 2000 to 2022, trends in the number of publications in HNSCC immunotherapy research **(A)**. Analysis of cooperation between countries **(B)**. Network of institutions engaged in HNSCC immunotherapy research **(C)**.

Annual growth trend of publications through a search of the WoSCC database, 1377 articles on immunotherapy for HNSCC published between 2000 and 2022 was identified. As shown in **Figure 2A**, the number of relevant research articles on this topic has exploded since 2016. The number of published articles increased from 31 in 2015 to 54 in 2016. This may be related to the approval in 2016 of the anti-programmed death-1 (PD-1) immune checkpoint inhibitors nivolumab and pembrolizumab for the treatment of HNSCC patients with relapse or metastasis during or after platinum-containing chemotherapy (5). The maximum number of articles published in 2022 is 301.

### Analysis of country and region output

A total of 69 countries or regions have provided all publications on immunotherapy for HNSCC. **Figure 2B** illustrates the network of national collaborations for HNSCC immunotherapy research. **Table 1** lists the ten countries that have the highest contribution. More than 100 publications have been provided in four countries or regions. The most significant contributor was the United States (538), followed by China (407), Germany (175), and Japan (119). Among the top 10 countries, the United States is a significant contributor to immunotherapy research for head and neck tumors, with 39% of the studies published. The centrality index measures the salience of network nodes (26). Centrality analysis shows that the United States (0.44) is the core of the network, which indicates that the United States acts as a middleman in the national cooperation network and has a great influence on publishing, followed by Germany (0.15). In a collaborative network (**Figure 2B**), higher centrality equates to more intensive cooperation. The chart of the country-based research network shows a low density, which means that the research team is relatively independent and emphasizes the need for further collaboration.

**Table 1:** Top 10 countries publishing research articles on immunotherapy for HNSCC.

Rank	Country	Publications	Citations	Centrality
1	USA	538	18180	0.44
2	PEOPLES R CHINA	407	4828	0.02
3	GERMANY	175	3939	0.15
4	JAPAN	119	2788	0.13
5	ITALY	63	973	0.06
6	FRANCE	48	1497	0.05
7	ENGLAND	47	1732	0.17
8	CANADA	39	1132	0.03
9	NETHERLANDS	35	1900	0.01
10	AUSTRALIA	34	806	0.04

### Institutions that publish clinical research papers on immunotherapy for HNSCC

The University of Pittsburgh (n=73), Sun Yat-sen University (n=60), Wuhan University (n=46), Johns Hopkins University (n=39), and the University of Texas MD Anderson Cancer Center(n=37) are among the top ten institutions for the number of published papers in head and neck cancer immunotherapy research (**Table 2**). The network map of the institutions engaged in the study reveals that the map density is low (Density=0.0099) (**Figure 2C**), suggesting that the research group is dispersed, the institutions are dispersed, and collaboration has to be increased. Most nodes have a centrality score of less than 0.15, suggesting that most institutions continue to have a low level of impact and that collaboration between institutions is insufficient.

**Table 2:** Top 10 institutions publishing research articles on immunotherapy for HNSCC.

Rank	Institution	Publications	Citations	Centrality
1	University of Pittsburgh	73	3011	0.18
2	Sun Yat-sen University	60	1005	0.08
3	Wuhan University	46	1232	0.01
4	Johns Hopkins University	39	1911	0.06
5	The University of Texas MD Anderson Cancer Center	37	1621	0.06
6	University of Michigan	34	1581	0.09
7	Dana-Farber Cancer Institute	27	1395	0.06
8	Memorial Sloan Kettering Cancer Center	25	2798	0.03
9	Baylor Coll Medical	24	864	0.02
10	University of Chicago	24	2848	0.12

### Analysis of the author and the co-cited author

**Figure 3:**
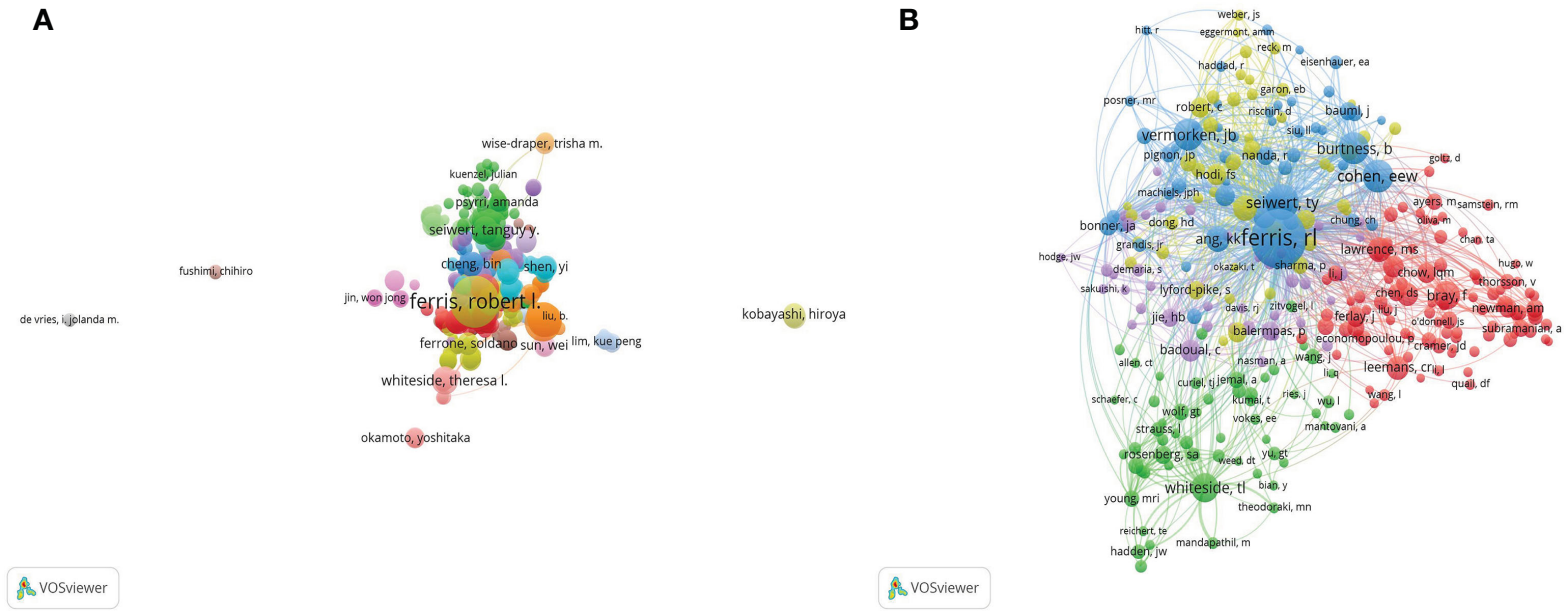
A network map showing authors **(A)** and co-cited authors **(B)** involved in immunotherapy research about HNSCC.

More than 700 researchers have participated in studies related to immunotherapy for HNSCC. Among them, the top three authors who have published the most papers are Robert L Ferris (n= 51), Zhijun Sun(n=24), and Zhang Wenfeng (n= 21) (**Table 3**). Among the top 10 co-citation authors (**Table 3**), FERRIS RL (n= 766) ranks first, followed by SEIWERT TY(n=281) and COHEN EEW (n= 240). The VOSviewer investigates co-authorship and citation networks between authors (**Figure 3A**). Each node on the chart represents each author, the size of the circle reflects the number of papers published by the researchers, and the lines connecting the circle represent the co-occurrence relationship between the authors. There is a close co-occurrence relationship between authors and co-citation authors, and prolific authors co-occur more with other authors (**Figure 3B**).

**Table 3:** Top 10 authors and co-cited authors involved in HNSCC immunotherapy research.

Rank	Author	Counts	Citations	Rank	Co-cited Author	Citations
1	Robert L Ferris	51	2305	1	Ferris RL	766
2	Sun Zhijun	24	987	2	Seiwert TY	281
3	Zhang Wenfeng	21	949	3	Cohen EEW	240
4	Deng Weiwei	19	823	4	Vermorken JB	228
5	Mao Liang	18	734	5	Burtness B	227
6	Yu Guangtao	17	826	6	Whiteside TL	182
7	Sikora G Andrew	17	758	7	Topalian SL	147
8	White L Theresa	16	615	8	Ang KK	141
9	Wu Lei	15	459	9	Lawrence MS	134
10	Seiwert Y Tanguy	14	1159	10	Gillison ML	123

### Analysis of journals and co-cited academic journals

**Figure 4:**
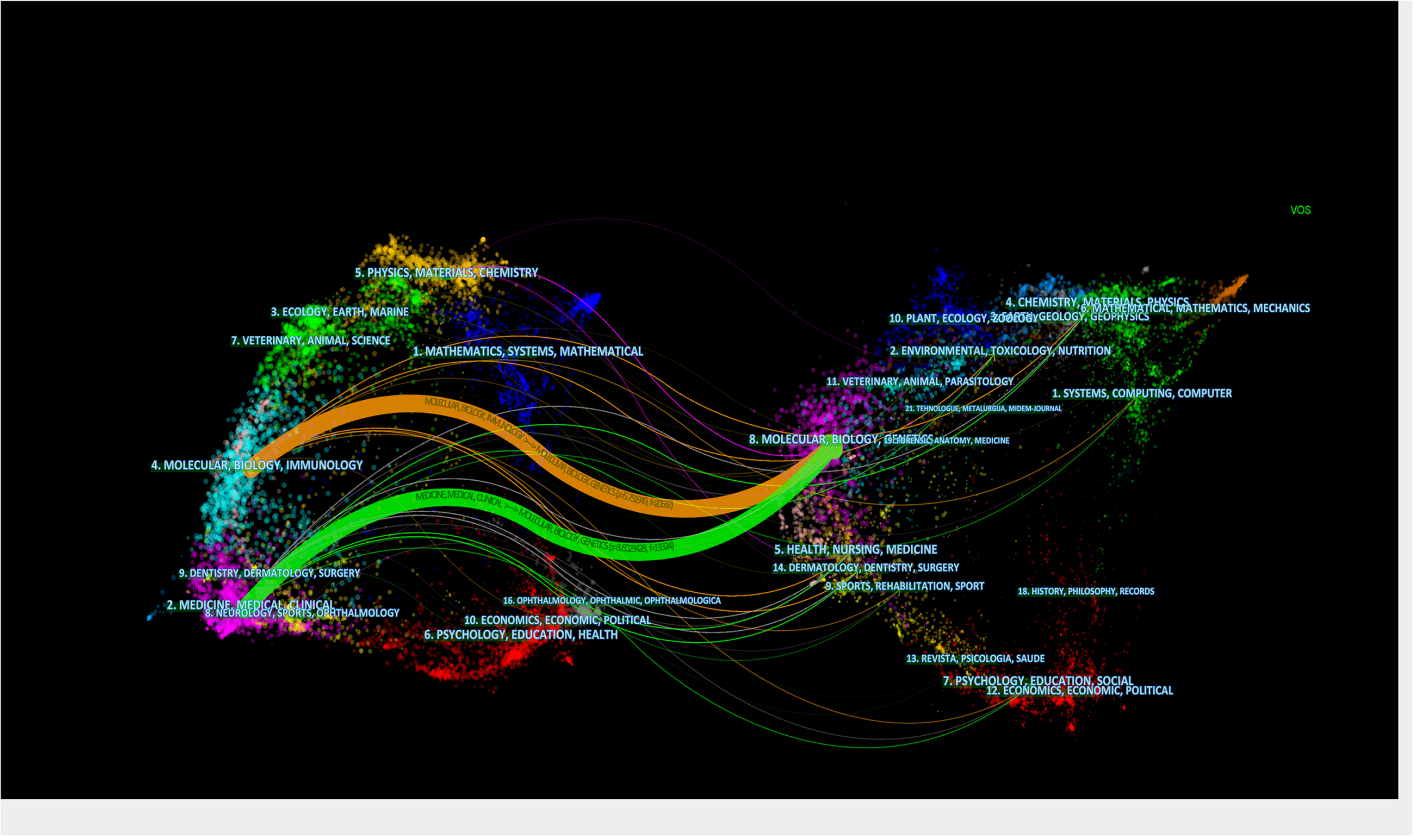
Dual-map overlay includes HNSCC immunotherapy-related publications.

In the research direction of immunotherapy for HNSCC, 546 academic journals have published related articles in this field. Among the ten most active journals, Oral Oncology (67 articles),Frontiers in Oncology (60 articles), Cancers (59 articles), Journal for Immunotherapy of Cancer (47 articles), Head and Neck-Journal for The Sciences And Specialties Of The Head And Neck (45 articles), and Clinical Cancer Research (42 articles) published more than 40 papers. Of the top 10 journals (**Table 4**), 40% of the journals are from the United States, followed by 30% from Switzerland. Clinical Cancer Research received the highest number of citations (915 citations). Notably, Cancer Research (870 citations) and the New England Journal of Medicine (808 citations) ranked 2nd and 3rd among co-cited journals. They are recognized as research resources for HNSCC immunotherapy. Most of the top 10 productive journals were related to immunology and clinical, while most of the 10 highly co-cited journals were related to oncology, which was consistent with the dual-map overlay analysis. A dual-map overlay was used to illustrate the subject distribution of academic journals (27). The dual-map overlay of the journal in **Figure 4** shows the topic distribution of the journal. The cited journal is on the left side of the map, while the cited journal is on the right side of the map. These labels represent the disciplines covered by the journal. From left to right, colored lines depict the reference path. There are two different citation paths. The orange citation path indicates that molecular/biological/genetic journals are cited by molecular/biological/immunological journals. The green approach shows that the research and application of molecular/biological/genetic are often cited by medicine/medical/clinical journals.

**Table 4:** Top 10 journals by several publications and co-citations.

Rank	Journal	Publications	Country	IF(2021)	Rank	Cited Journal	Citations	Country	IF(2021)
1	ORAL ONCOLOGY	67	ENGLAND	5.972	1	CLINICAL CANCER RESEARCH	915	USA	13.801
2	FRONTIERS IN ONCOLOGY	60	SWITZERLAND	5.738	2	CANCER RESEARCH	870	USA	13.312
3	CANCERS	59	SWITZERLAND	6.575	3	NEW ENGLAND JOURNAL OF MEDICINE	808	USA	176.079
4	JOURNAL FOR IMMUNOTHERAPY OF CANCER	47	ENGLAND	12.469	4	JOURNAL OF CLINIC ONCOLOGY	783	USA	50.717
5	HEAD AND NECK-JOURNAL FOR THE SCIENCES AND SPECIALTIES OF THE HEAD AND NECK	45	USA	3.821	5	NATURE	634	ENGLAND	69.504
6	CLINICAL CANCER RESEARCH	42	USA	13.801	6	ORAL ONCOLOGY	585	ENGLAND	5.972
7	CANCER IMMUNOLOGY IMMUNOTHERAPY	39	USA	6.63	7	THE JOURNAL OF IMMUNOLOGY	515	USA	5.426
8	ONCOIMMUNOLOGY	37	USA	7.723	8	INTERNATIONAL JOURNAL OF CANCER	510	SWITZERLAND	7.316
9	FRONTIERS IN IMMUNOLOGY	35	SWITZERLAND	8.786	9	PROCEEDINGS OF THE NATIONAL ACADEMY OF SCIENCES OF THE UNITED STATES OF AMERICA	496	USA	12.779
10	HNO	31	GERMANY	1.33	10	LANCET ONCOLOGY	477	USA	54.433

### Analysis of co-cited references

Two or more references cited by one or more papers simultaneously constitute a typical citation relationship, and two or more connections are commonly cited references (28). The analysis of publications with high co-citation frequency helps understand the basis of disciplinary research. **Table 5** summarizes the top ten co-cited references. The most frequently cited article on the research of immunotherapy for HNSCC is the Nivolumab for Recurrent Squamous-Cell Carcinoma of the Head and Neck (29). This article was published by Ferris RL in the New England Journal of Medicine and was cited 395 times. The literature with a total co-citation number of more than 200(n=3) was composed of articles published in the New England Journal of Medicine, Lancet Oncology, and Lancet.

**Table 5:** Top 10 Most Cited References in Immunotherapy for HNSCC.

Rank	Cited frequency	Title	Author	Year	Journal	IF(2021)
1	395	Nivolumab for recurrent squamous-cell carcinoma of the head and neck	Ferris RL	2016	NEW ENGLAND JOURNAL OF MEDICINE	176.079
2	219	Safety and clinical activity of pembrolizumab for treatment of recurrent or metastatic squamous cell carcinoma of the head and neck (KEYNOTE-012): an open-label, multicentre, phase 1b trial	Seiwert TY	2016	LANCET ONCOLOGY	54.433
3	203	Pembrolizumab alone or with chemotherapy versus cetuximab with chemotherapy for recurrent or metastatic squamous cell carcinoma of the head and neck (KEYNOTE-048): a randomised, open-label, phase 3 study	Burtness B	2019	LANCET	202.731
4	143	Pembrolizumab versus methotrexate, docetaxel, or cetuximab for recurrent or metastatic head-and-neck squamous cell carcinoma (KEYNOTE-040): a randomised, open-label, phase 3 study	Cohen EEW	2019	LANCET	202.731
5	140	Global cancer statistics 2018: GLOBOCAN estimates of incidence and mortality worldwide for 36 cancers in 185 countries	Freddie Bray	2018	CA-A CANCER JOURNAL FOR CLINICIANS	286.13
6	111	Comprehensive genomic characterization of head and neck squamous cell carcinomas	Lawrence MS	2015	NATURE	69.504
7	109	Immunology and immunotherapy of head and neck cancer	Ferris RL	2015	JOURNAL OF CLINIC ONCOLOGY	50.717
8	99	Pembrolizumab in patients with advanced triple-negative breast cancer: phase Ib KEYNOTE-012 study	Nanda R	2016	JOURNAL OF CLINIC ONCOLOGY	50.717
9	97	The head and neck cancer immune landscape and its immunotherapeutic implications	Mandal R	2016	JCI INSIGHT	9.484
10	76	Pembrolizumab for platinum- and cetuximab-refractory head and neck cancer: results from a single-arm, phase ii study	Bauml J	2017	JOURNAL OF CLINIC ONCOLOGY	50.717

**Figure 5:**
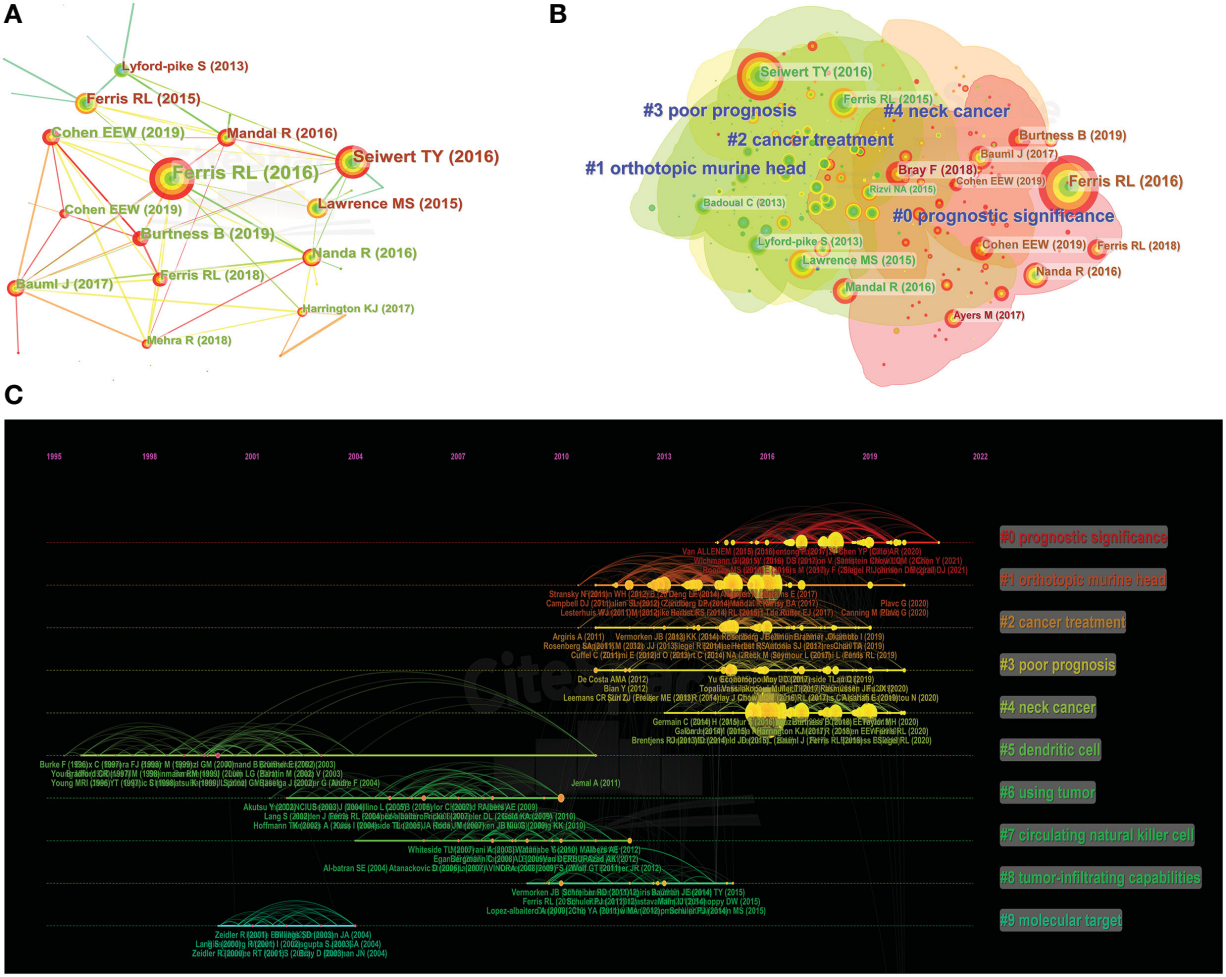
Co-cited references map **(A)** and clustered network map of co-cited references **(B)** on HNSCC immunotherapy research from 2000 to 2022. Each node’s diameter is proportional to the total number of co-citations for the connected article. The broad red circles outside the nodes suggested a high degree of centrality. The timeline view of the clusters that were co-cited along with their labels **(C)**. The nodes on the left represent older references, while the nodes on the right represent more recent references. A straight line in the same horizontal position indicates the set of all clustered references belonging, and the cluster label is located at the line’s rightmost end.

CiteSpace was used to assess the references with a high citation burst. Citation bursts indicated that a reference had been widely cited over time and that the study findings of the references were well-known in this field. In this study, a co-citation correlation analysis was performed on 25343 cited references from 1377 publications, resulting in a clustering network diagram. **Figure 5A** shows a visual network of co-cited references. Each node represents a cited article. The frequency with which the same article is cited is represented by links between nodes. The diameter of the node is proportional to the total number of citations. The top ten references cited are mainly concentrated in the clustering of prognostic significance, orthotopic murine head, cancer treatment, and poor prognosis (**Figure 5B**). There were ten main clusters of co-cited references, consisting of prognostic significance, orthotopic murine head, cancer treatment, poor prognosis, neck cancer, dendritic cell, using tumor, circulating natural killer cell, tumor-infiltrating capabilities, and molecular target. The timeline view is a methodology for visualizing data that combines clustering and time-slicing techniques (30). **Figure 5C** shows a timeline view of the clustering diagram, which supports the findings of developing research hotspots in HNSCC immunotherapy.

### High-frequency keyword analysis

**Figure 6:**
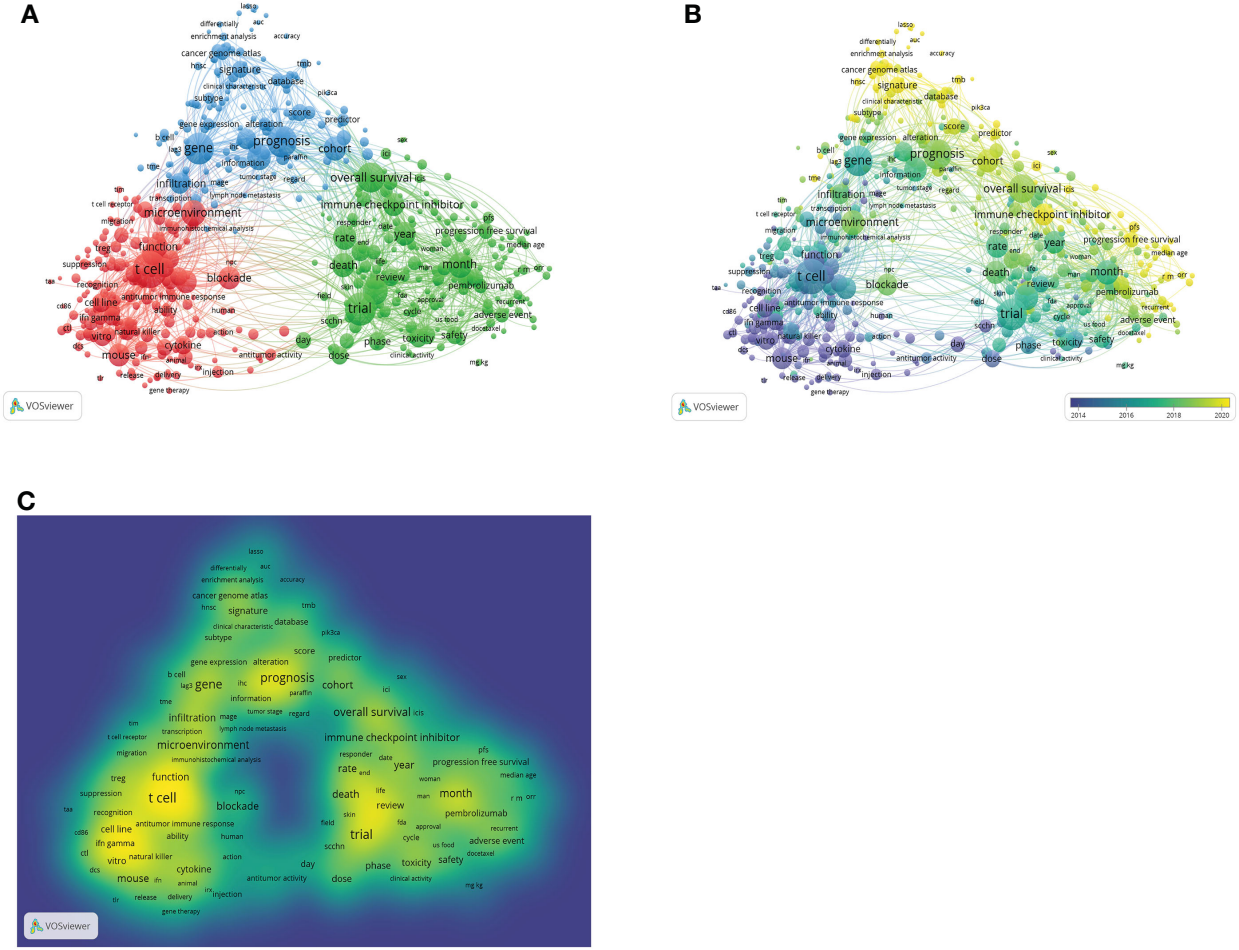
Bibliometric analysis of keywords in research papers on immunotherapy of HNSCC **(A)** Mapping of keywords in the HNSCC immunotherapy research. Thematic network maps of trends based on keywords used from January 2000 to December 2022 **(B)**. The indicator shows the current publication from purple to yellow. The size of the circle indicates the frequency of occurrence, and the distance between the two circles indicates the correlation between them. Keyword density map **(C)**. The most frequently used keywords are denoted by the color yellow.

Keywords are the core of the article (16). Through the analysis of the article’s keywords, we can peep into the theme of the article. By analyzing keywords, hot spots in specific research fields can be found (31, 32). The VOSviewer is used to see the network of keywords, as illustrated in **Figure 6A**. Three study directions are indicated by the clustering of red, blue, and green. The red category is mostly concerned with the investigation of immune cell function in HNSCC immunotherapy which contains the keywords: t cell, blockage, function, microenvironment, and natural killer. Topics in the blue category include gene, prognosis, cohort, cancer genome, infiltration, and predictors. This group is primarily concerned with the prognosis of HNSCC patients who have received immunotherapy. Green cluster keywords include overall survival rate, recurrence, test, immunological checkpoint, toxicity, and safety. This cluster focuses primarily on the safety and toxicity of immunotherapeutic medicines, as well as their impact on patient survival. As illustrated in **Figure 6B**, the VOSviewer displays that purple signifies a very early appearance of the word, while yellow indicates a more recent appearance, depending on the period in which each word appears. In **Figure 6C**, the VOSviewer colors all keywords according to their average number of occurrences. For instance, T cells, prognosis, blockade, and trial are often used as key terms, which shows the shift in focus and direction of future research to some extent.

## Discussion

### General information

In this study, CiteSpace and VOSviewer were utilized to conduct a bibliometric examination of the scientific results of immunotherapy for HNSCC published worldwide from 2000 to 2022. The bibliometric analysis contains 1377 English-language articles published in 397 publications by 546 organizations in 69 countries. The records received from the WoSCC are evaluated from a variety of perspectives, and the results are displayed as tables and knowledge network diagrams. The United States provided 538 publications among the top 10 publishing nations, demonstrating its dominance in HNSCC immunotherapy research. China follows with 407 articles published. In terms of the number of publications and centrality, institutions with strong scientific research focus mostly on higher education research institutes, which serve as the foundation for medical research and education. The top ten universities published 389 publications. Eight of the universities or institutions on this list are located in the United States. They are the University of Pittsburgh, Johns Hopkins University, the University of Texas MD Anderson Cancer Center, the University of Michigan, Dana-Farber Cancer Institute, Memorial Sloan Kettering Cancer Center, Baylor Coll Medical, and the University of Chicago. This indicates that universities and institutions in the United States have made total contributions and comparative research in the field of HNSCC immunotherapy. And the remaining two universities Sun Yat-sen University and Wuhan University are located in China. The institution distribution table provides useful information that will assist researchers in identifying and selecting appropriate partner institutions. Robert L Ferris has produced the most publications in the field of HNSCC immunotherapy. Furthermore, Sun Zhijun, Zhang Wenfeng, Deng Weiwei, and Mao Liang were the top five most productive authors of the past 22 years in this research field. It is important to note that the majority of HNSCC immunotherapy researchers work in the United States and China. Therefore, enhancing global researchers’ communication and cooperation would facilitate the production of HNSCC immunotherapy research. When co-cited authors are included, the top 10 HNSCC immunotherapy authors with at least 123 co-citations have made substantial contributions. Oral Oncology (67) ranked first in terms of total publications, followed by Frontiers in Oncology (60) and Cancers (59), showing that these journals were most interested in HNSCC immunotherapy research articles. These statistics will assist future researchers in selecting journals to which to submit publications on immunotherapy for HNSCC. Researchers are more concerned with the clinical management of HNSCC immunotherapy, according to the top ten co-cited references from 2000 to 2022. The first reference with the highest number of co-citations and a landmark was the article “Nivolumab for Recurrent Squamous-Cell Carcinoma of the Head and Neck” published by Ferris RL (29), which proposed that treatment with nivolumab resulted in longer overall survival than standard, single-agent therapy in patients with platinum-refractory, recurrent squamous-cell carcinoma of the head and neck. The first three referenced publications in Clinical Cancer Research, Cancer Research, and New England Journal of Medicine discuss immunotherapy in patients with recurrent/metastatic (R/M) HNSCC, and five of the top ten cited articles were connected to immunotherapy with the drug pembrolizumab. In patients with R/M HNSCC who progressed during or after platinum chemotherapy, treatment with the programmed cell death 1 (PD-1) inhibitors pembrolizumab and nivolumab increased overall survival compared to conventional therapy (29, 33). Most of the references are gathered in the first five clusters of prognostic significance, orthotopic murine head, cancer treatment, poor prognosis, and neck cancer. According to the review of references, the hot point of immunotherapy for head and neck cancers in recent years has been mostly focused on the prognosis of patients with squamous-cell carcinoma of head and neck. These ten frequently co-cited references illustrated the present epidemiology of HNSCC, the study and development of immunotherapy for R/M HNSCC, and the clinical use of these treatments. Co-citation analysis can provide a plethora of relevant information, enabling us to acquire a deeper understanding of the evolution of the knowledge structure about HNSCC immunotherapy. The vast majority of research publications on HNSCC immunotherapy were published in the molecular/biological/genetic field, according to references. Researchers from a wide range of fields, including molecular biology and genetics, will be needed to develop the best immunotherapy for HNSCC.

### Research priorities and frontiers

CiteSpace was utilized to analyze the co-cited references to study and characterize the new focus of immunotherapy for HNSCC. Early studies, as depicted in **Figure 5C**, focused on the molecular basis and molecular target of immunotherapy for head and neck squamous cell carcinoma, such as “# 5 dendritic cell”, “# 7 circulating natural killer cell”, and “# 9 molecular target”, whereas current studies have emphasized “# 0 prognostic significance”, “# 2 cancer treatment”, and “# 3 poor prognosis.” At the same time, the present phase focuses on determining how to evaluate the efficacy indicators and prognosis of immunotherapy patients. Immunotherapy has been proposed as a complementary treatment for HNSCC and other forms of cancer. Immune cells, including tumor-infiltrating lymphocytes, T cells, plasma cells, B cells, macrophages, neutrophils, monocytes, and dendritic cells, invade HNSCC extensively. Dendritic cells (DCs) have the unique capacity to carry tumor antigens to drain lymph nodes to activate T cells, which is essential for T cells to rely on immunity and respond to immune checkpoint blockade (ICB) (34, 35). DCs are the most significant cell type integrating the innate and adaptive immune systems, and they play an important role in inflammation by initiating and polarizing immune responses (9). Immature DCs patrol tissues, and recognize, and capture antigens, causing them to mature. Matured DCs release inflammatory mediators, have a high potential for T-cell stimulation and have antigen presentation properties (9, 36, 37). Due to their function in producing adaptive protective immunity, DCs have been a focus of cancer immunotherapy for decades (38–41). DCs have a crucial role in regulating the tumor microenvironment and immune response during HNSCC progression (5, 39). Natural killer (NK) cells are an important part of the innate immune system because they are the first line of defense against cancer cells. Thus, they might serve as a predictive biomarker in HNSCC (42). The classic phenotypic marker of NK cells, CD56, has been identified in the majority of research (42, 43). NK cells have an innate origin, but they also perform adaptive immune tasks. Due to their propensity to lyse tumor cells lacking appropriate levels of MHC class 1, NK cells are attractive candidates for prospective biomarkers, as the immune system is a critical factor in tumor growth and control (42, 44). The two clusters of “# 0 prognostic significance” and “# 3 poor prognosis” suggest that the focus of immunotherapy for head and neck cancers in recent years has been on overall prognosis. Late-stage HNSCC has a poor prognosis, with a relatively low survival rate (45). Since tumor cells evade immunosurveillance by activating inhibitory checkpoint pathways that limit anti-tumor T-cell responses, accumulating scientific evidence suggests that the immune system plays a crucial role in the development of HNSCC (46). Immunotherapy is an effective treatment for HNSCC patients. Immunotherapy aims to boost the immune system’s ability to remove cancer cells (47). Immune checkpoint inhibitors (ICIs) are a type of immunotherapy that can disrupt the IC inhibition pathway and enhance anti-tumor immunity. Anti-PD-1/PD-L1 ICIs can strengthen anti-tumor immunity. ICIs had previously been approved for a variety of solid tumors. In 2017, nivolumab was approved as a second-line therapy for platinum-resistant R/M HNSCC (48). Even though ICIs has a good therapeutic impact on HNSCC, the response rate is still relatively low (49). As a result, identifying biomarkers that predict ICI response is useful for patient screening and customized treatment, and it is critical for standardizing immunosuppressive medication and improving the prognosis of patients with HNSCC (50).

In addition, we used VOSviewer to evaluate keywords, which may be indicative of a future research hotspot or frontier. As depicted in **Figure 6B**, the development of keywords indicates that immunotherapy for head and neck cancers continues to progress. Similar to the findings of the co-cited references, we discovered that immunotherapy for HNSCC has recently focused more on the prognosis of tumor patients, as evidenced by the keywords “prognosis”, “overall survival”, and “progression free survival” in the study of the annual progress of keywords. The introduction of ICIs targeting the PD-1/PD-L1 pathway in recent years has resulted in further breakthroughs in the fate of patients with metastatic HNSCC, although the results remain unsatisfactory when compared to other tumors such as melanoma and lung cancer (51). For patients with HNSCC treated with surgery, chemoradiotherapy, radiation, or mixed therapies, elevated PD-L1 expression has been linked to a poorer prognosis in the majority of studies examining its prognostic value (52). However, research indicates that immunotherapy may also benefit some PD-L1-negative patients (53). Tumor mutational burden (TMB) is significantly connected with the overall response rate to anti-PD-1 or anti-PD-L1 therapy, as demonstrated by a variety of researches (54). It is believed that elevated TMB provides a significant number of novel antigens, hence enhancing the immunogenicity of the tumor and enhancing the response to checkpoint inhibition (47). Additional research has indicated that elevated TMB is associated with enhanced response and survival in various malignant tumors, including non-small cell lung cancer (NSCLC) (55). HNSCC development and progression are significantly influenced by the host’s immune responses. In recent years, the importance of the tumor microenvironment (TME), particularly the tumor immune microenvironment, in HNSCC has been increasingly evident. It has been shown that recurrent HNSCC is characterized by TME that is immunosuppressive and possesses complicated immune escape mechanisms (56).In addition, tumor-infiltrating lymphocytes(TILs), which are modified by the TME, have been shown to represent independent prognostic variables in HNSCC (39, 57). The results on TMB and TILs as prognostic biomarkers for immunotherapy in HNSCC are promising, but the routine assessment of these biomarkers in everyday practice is still challenging, and they require validation through prospective investigations. To increase the efficacy of the immune response and the prognosis of patients, present and future research are being conducted to determine an overview of the immune response, which includes biomarkers and clinical aspects.

Bibliometrics is a method for describing the evolution of scientific knowledge and its structural relationship, which reveals numerous hidden complicated links between clusters of information (20, 25). Therefore, by comprehending these intricate knowledge relationships, scholars can comprehend specific knowledge trends. In recent years, tumor immunotherapy has become a relatively novel and promising treatment. Although this treatment can provide considerable clinical outcomes, tumor heterogeneity and diversity are unavoidable. Immunotherapy or combination therapy may not be beneficial for all HNSCC patients due to their varied immunological environments (7, 11, 58). By comprehending these intricate knowledge links, scholars can comprehend specific knowledge patterns. Finding more effective immune targets, elucidating the immune characteristics of the immune microenvironment of head and neck cancers, and determining the survival and prognostic indicators of immunotherapy patients may play an important role in tumor immunotherapy over the next few years based on our bibliometric analysis.

## Conclusion

In recent years, the incidence of HNSCC has been steadily increasing, hence immunotherapy of HNSCC is of critical importance. This paper summarizes and analyzes the trend in immunotherapy research for HNSCC using the bibliometrics method. Our investigation revealed an upward trend in the total number of posts, indicating that researchers are becoming increasingly interested in this promising field. In recent years, prognosis, overall survival, and progression-free survival have been the focal points of study in this field, showing that the direction of research is concentrated on the prognosis of immunotherapy in patients with HNSCC.

## Strengths and limitations

To the best of our knowledge, this is the first comprehensive review of HNSCC immunotherapy articles and trends that provides physicians and scholars in the area with detailed information. In addition, we used a number of bibliometric software to examine research hotspots in a variety of ways. This study is bound to have some flaws. To begin, it is possible that the research we used wasn’t exhaustive. On the one hand, our analysis focused on data from WoSCC, excluding data from other prominent search engines including PubMed, Embase, and Ovid. However, there is a linguistic bias created because the retrieved articles are limited to publications published in English. These studies may not accurately represent all immunotherapy trials for HNSCC. Second, because citation rates are so low, recently published, high-quality publications may not get the attention they deserve. This shows the importance of future research updates, as evidenced by this example. As a final point, even though this study only examined papers published between 2000 and 2022 in the field of HNSCC immunotherapy, eliminating documents from prior years and publications in 2023, fresh data may have consequences for the results. Co-occurrence and co-citation analysis methods for bibliometric and visual depiction of immunotherapy in HNSCC have never been used in a study before. There are some drawbacks to this study. Only English-language papers were evaluated in WoSCC because of technical restrictions. Therefore, our findings may not apply to studies published in other languages, and our data may not be thorough. When keywords are grouped, different types of content may end up with some overlap.

## Data availability statement

The original contributions presented in the study are included in the article/supplementary material. Further inquiries can be directed to the corresponding author.

## Author contributions

JW and MY designed the study. JW interpreted the data and wrote the article.MY collected and analyzed the data. checked the data. ZG and MY revised the article. All authors read and approved the final manuscript.

## References

[1] Bray F Ferlay J Soerjomataram I Siegel RL Torre LA Jemal A. Global cancer statistics 2018: GLOBOCAN estimates of incidence and mortality worldwide for 36 cancers in 185 countries. CA Cancer J Clin (2018) 68(6):394–424. doi: 10.3322/caac.21492 30207593

[2] Mody MD Rocco JW Yom SS Haddad RI Saba NF. Head and neck cancer. Lancet (2021) 398(10318):2289–99. doi: 10.1016/s0140-6736(21)01550-6 34562395

[3] Kao HF Lou PJ. Immune checkpoint inhibitors for head and neck squamous cell carcinoma: Current landscape and future directions. Head Neck (2019) 41 Suppl 1:4–18. doi: 10.1002/hed.25930 31573752

[4] Muzaffar J Bari S Kirtane K, Chung CH. Recent advances and future directions in clinical management of head and neck squamous cell carcinoma. Cancers (Basel) (2021) 13(2):338. doi: 10.3390/cancers13020338 33477635 PMC7831487

[5] Mei Z Huang J Qiao B, Lam AK. Immune checkpoint pathways in immunotherapy for head and neck squamous cell carcinoma. Int J Oral Sci (2020) 12(1):16. doi: 10.1038/s41368-020-0084-8 PMC7253444 32461587

[6] Bhatia A Burtness B. Treating head and neck cancer in the age of immunotherapy: A 2023 update. Drugs (2023). doi: 10.1007/s40265-023-01835-2 36645621

[7] Duray A Demoulin S Hubert P Delvenne P and Saussez S. Immune suppression in head and neck cancers: a review. Clin Dev Immunol (2010) 2010):701657. doi: 10.1155/2010/701657 21437225 PMC3061296

[8] Yao Y Yan Z Lian S Wei L Zhou C Feng D. Prognostic value of novel immune-related genomic biomarkers identified in head and neck squamous cell carcinoma. J Immunother Cancer (2020) 8(2):e000444. doi: 10.1136/jitc-2019-000444 32719094 PMC7390201

[9] Hoffmann C Noel F Grandclaudon M Massenet-Regad L Michea P Sirven P. PD-L1 and ICOSL discriminate human secretory and helper dendritic cells in cancer, allergy and autoimmunity. Nat Commun (2022) 13(1):1983. doi: 10.1038/s41467-022-29516-w 35418195 PMC9008048

[10] Badoual C Hans S Merillon N Van Ryswick C Ravel P Benhamouda N. PD-1-expressing tumor-infiltrating T cells are a favorable prognostic biomarker in HPV-associated head and neck cancer. Cancer Res (2013) 73(1):128–38. doi: 10.1158/0008-5472.Can-12-2606 23135914

[11] Beatty GL Gladney WL. Immune escape mechanisms as a guide for cancer immunotherapy. Clin Cancer Res (2015) 21(4):687–92. doi: 10.1158/1078-0432.CCR-14-1860 PMC4334715 25501578

[12] Cramer JD Burtness B and Ferris RL. Immunotherapy for head and neck cancer: Recent advances and future directions. Oral Oncol (2019) 99:104460. doi: 10.1016/j.oraloncology.2019.104460 31683169 PMC7749717

[13] Rothschild U Muller L Lechner A Schlosser HA Beutner D Laubli H. Immunotherapy in head and neck cancer - scientific rationale, current treatment options and future directions. Swiss Med Wkly (2018) 148:w14625. doi: 10.4414/smw.2018.14625 29756633

[14] Moskovitz J Moy J and Ferris RL. Immunotherapy for head and neck squamous cell carcinoma. Curr Oncol Rep (2018) 20(2):22. doi: 10.1007/s11912-018-0654-5 29502288 PMC5835060

[15] Wang J Liang S Yu M and Gong Z. COVID-19 from the perspective of otorhinolaryngology: An analysis of bibliometrics. Front Public Health (2022) 10:1002686. doi: 10.3389/fpubh.2022.1002686 36211675 PMC9539910

[16] van Eck NJ Waltman L. Citation-based clustering of publications using CitNetExplorer and VOSviewer. Scientometrics (2017) 111(2):1053–70. doi: 10.1007/s11192-017-2300-7 PMC5400793 28490825

[17] Huang T Wu H Yang S Su B Tang K Quan Z. Global trends of researches on sacral fracture surgery: A bibliometric study based on VOSviewer. Spine (Phila Pa 1976) (2020) 45(12):E721–e8. doi: 10.1097/brs.0000000000003381 31972744

[18] Chen C Hu Z Liu S and Tseng H. Emerging trends in regenerative medicine: a scientometric analysis in CiteSpace. Expert Opin Biol Ther (2012) 12(5):593–608. doi: 10.1517/14712598.2012.674507 22443895

[19] Gao Y Shi S Ma W Chen J Cai Y Ge L. Bibliometric analysis of global research on PD-1 and PD-L1 in the field of cancer. Int Immunopharmacol (2019) 72:374–84. doi: 10.1016/j.intimp.2019.03.045 31030093

[20] Xie L Chen Z Wang H Zheng C and Jiang J. Bibliometric and visualized analysis of scientific publications on atlantoaxial spine surgery based on web of science and VOSviewer. World Neurosurg (2020) 137:435–42.e4. doi: 10.1016/j.wneu.2020.01.171 32006737

[21] Zhong D Luo S Zheng L Zhang Y and Jin R. Epilepsy occurrence and circadian rhythm: A bibliometrics study and visualization analysis *via* CiteSpace. Front Neurol (2020) 11:984. doi: 10.3389/fneur.2020.00984 33250835 PMC7674827

[22] Ke L Lu C Shen R Lu T Ma B and Hua Y. Knowledge mapping of drug-induced liver injury: A scientometric investigation (2010-2019). Front Pharmacol (2020) 11:842. doi: 10.3389/fphar.2020.00842 32581801 PMC7291871

[23] Chow LQM. Head and neck cancer. N Engl J Med (2020) 382(1):60–72. doi: 10.1056/NEJMra1715715 31893516

[24] Wu M Wang Y Yan C Zhao Y. Study on subclinical hypothyroidism in pregnancy: a bibliometric analysis *via* CiteSpace. J Matern Fetal Neonatal Med (2022) 35(3):556–67. doi: 10.1080/14767058.2020.1729731 32106735

[25] Chen C. CiteSpace II: Detecting and visualizing emerging trends and transient patterns in scientific literature. J Am Soc Inf Sci Technol (2006) 57(3):359–77. doi: 10.1002/asi.20317

[26] Wang R Weng LM Peng MS Wang XQ. Exercise for low back pain: A bibliometric analysis of global research from 1980 to 2018. J Rehabil Med (2020) 52(4):jrm00052. doi: 10.2340/16501977-2674 32296852

[27] Zhang J Song L Xu L Fan Y Wang T Tian W. Knowledge domain and emerging trends in ferroptosis research: A bibliometric and knowledge-map analysis. Front Oncol (2021) 11:686726. doi: 10.3389/fonc.2021.686726 34150654 PMC8209495

[28] Wu H Zhou Y Wang Y Tong L Wang F Song S. Current state and future directions of intranasal delivery route for central nervous system disorders: A scientometric and visualization analysis. Front Pharmacol (2021) 12:717192. doi: 10.3389/fphar.2021.717192 34322030 PMC8311521

[29] Ferris RL Blumenschein G Jr. Fayette J Guigay J Colevas AD Licitra L. Nivolumab for recurrent squamous-cell carcinoma of the head and neck. N Engl J Med (2016) 375(19):1856–67. doi: 10.1056/NEJMoa1602252 PMC5564292 27718784

[30] Logan AC Katzman M. Major depressive disorder: probiotics may be an adjuvant therapy. Med Hypotheses (2005) 64(3):533–8. doi: 10.1016/j.mehy.2004.08.019 15617861

[31] Wu H Cheng K Guo Q Yang W Tong L Wang Y. Mapping knowledge structure and themes trends of osteoporosis in rheumatoid arthritis: A bibliometric analysis. Front Med (Lausanne) (2021) 8:787228. doi: 10.3389/fmed.2021.787228 34888333 PMC8650090

[32] Gao Q Zhang C Wang J Wei Q Wei Q Miyamoto A. The top 100 highly cited articles on osteoporosis from 1990 to 2019: a bibliometric and visualized analysis. Arch Osteoporos (2020) 15(1):144. doi: 10.1007/s11657-020-0705-z 32935223

[33] Burtness B Harrington KJ Greil R Soulières D Tahara M de Castro G. Pembrolizumab alone or with chemotherapy versus cetuximab with chemotherapy for recurrent or metastatic squamous cell carcinoma of the head and neck (KEYNOTE-048): a randomised, open-label, phase 3 study. Lancet (2019) 394(10212):1915–28. doi: 10.1016/s0140-6736(19)32591-7 31679945

[34] Broz ML Binnewies M Boldajipour B Nelson AE Pollack JL Erle DJ. Dissecting the tumor myeloid compartment reveals rare activating antigen-presenting cells critical for T cell immunity. Cancer Cell (2014) 26(5):638–52. doi: 10.1016/j.ccell.2014.09.007 PMC4254577 25446897

[35] Hildner K Edelson BT Purtha WE Diamond M Matsushita H Kohyama M. Batf3 deficiency reveals a critical role for CD8alpha+ dendritic cells in cytotoxic T cell immunity. Science (2008) 322(5904):1097–100. doi: 10.1126/science.1164206 PMC2756611 19008445

[36] Gaurav R Agrawal DK. Clinical view on the importance of dendritic cells in asthma. Expert Rev Clin Immunol (2013) 9(10):899–919. doi: 10.1586/1744666x.2013.837260 24128155 PMC4479275

[37] Li Q Guo Z Xu X Xia S and Cao X. Pulmonary stromal cells induce the generation of regulatory DC attenuating T-cell-mediated lung inflammation. Eur J Immunol (2008) 38(10):2751–61. doi: 10.1002/eji.200838542 18825748

[38] Gardner A de Mingo Pulido A Ruffell B. Dendritic cells and their role in immunotherapy. Front Immunol (2020) 11:924. doi: 10.3389/fimmu.2020.00924 32508825 PMC7253577

[39] Fan C Wu J Shen Y Hu H Wang Q Mao Y. Hypoxia promotes the tolerogenic phenotype of plasmacytoid dendritic cells in head and neck squamous cell carcinoma. Cancer Med (2022) 11(4):922–30. doi: 10.1002/cam4.4511 PMC8855917 34964283

[40] Dudek AM Martin S Garg AD Agostinis P. Immature, semi-mature, and fully mature dendritic cells: Toward a DC-cancer cells interface that augments anticancer immunity. Front Immunol (2013) 4:438. doi: 10.3389/fimmu.2013.00438 24376443 PMC3858649

[41] Lu S Concha-Benavente F Shayan G Srivastava RM Gibson SP Wang L. STING activation enhances cetuximab-mediated NK cell activation and DC maturation and correlates with HPV(+) status in head and neck cancer. Oral Oncol (2018) 78:186–93. doi: 10.1016/j.oraloncology.2018.01.019 PMC5877820 29496049

[42] Bisheshar SK De Ruiter EJ Devriese LA Willems SM. The prognostic role of NK cells and their ligands in squamous cell carcinoma of the head and neck: a systematic review and meta-analysis. Oncoimmunology (2020) 9(1):1747345. doi: 10.1080/2162402x.2020.1747345 32363116 PMC7185215

[43] Poli A Michel T Thérésine M Andrès E Hentges F and Zimmer J. CD56bright natural killer (NK) cells: an important NK cell subset. Immunology (2009) 126(4):458–65. doi: 10.1111/j.1365-2567.2008.03027.x PMC2673358 19278419

[44] Solana R Tarazona R Gayoso I Lesur O Dupuis G and Fulop T. Innate immunosenescence: effect of aging on cells and receptors of the innate immune system in humans. Semin Immunol (2012) 24(5):331–41. doi: 10.1016/j.smim.2012.04.008 22560929

[45] Johnson DE Burtness B Leemans CR Lui VWY Bauman JE. And grandis J r. head and neck squamous cell carcinoma. Nat Rev Dis Primers (2020) 6(1):92. doi: 10.1038/s41572-020-00224-3 33243986 PMC7944998

[46] Economopoulou P Perisanidis C Giotakis EI Psyrri A. The emerging role of immunotherapy in head and neck squamous cell carcinoma (HNSCC): anti-tumor immunity and clinical applications. Ann Transl Med (2016) 4(9):173. doi: 10.21037/atm.2016.03.34 27275486 PMC4876265

[47] Gavrielatou N Doumas S Economopoulou P Foukas PG Psyrri A. Biomarkers for immunotherapy response in head and neck cancer. Cancer Treat Rev (2020) 84:101977. doi: 10.1016/j.ctrv.2020.101977 32018128

[48] Kitamura N Sento S Yoshizawa Y Sasabe E Kudo Y, Yamamoto T. Current trends and future prospects of molecular targeted therapy in head and neck squamous cell carcinoma. Int J Mol Sci (2020) 22(1):240. doi: 10.3390/ijms22010240 33383632 PMC7795499

[49] Yi L Wu G Guo L Zou X and Huang P. Comprehensive analysis of the PD-L1 and immune infiltrates of m(6)A RNA methylation regulators in head and neck squamous cell carcinoma. Mol Ther Nucleic Acids (2020) 21:299–314. doi: 10.1016/j.omtn.2020.06.001 32622331 PMC7332506

[50] Guo Y Pan WK Wang ZW Su WH Xu K Jia H. Identification of novel biomarkers for predicting prognosis and immunotherapy response in head and neck squamous cell carcinoma based on ceRNA network and immune infiltration analysis. BioMed Res Int (2021) 2021:4532438. doi: 10.1155/2021/4532438 34917682 PMC8670464

[51] Chow LQM Haddad R Gupta S Mahipal A Mehra R Tahara M. Antitumor activity of pembrolizumab in biomarker-unselected patients with recurrent and/or metastatic head and neck squamous cell carcinoma: Results from the phase ib KEYNOTE-012 expansion cohort. J Clin Oncol (2016) 34(32):3838–45. doi: 10.1200/jco.2016.68.1478 PMC6804896 27646946

[52] Ngamphaiboon N Chureemas T Siripoon T Arsa L Trachu N Jiarpinitnun C. Characteristics and impact of programmed death-ligand 1 expression, CD8+ tumor-infiltrating lymphocytes, and p16 status in head and neck squamous cell carcinoma. Med Oncol (2019) 36(2):21. doi: 10.1007/s12032-018-1241-1 30666437

[53] Ferris RL Blumenschein G Jr. Fayette J Guigay J Colevas AD Licitra L. Nivolumab vs investigator's choice in recurrent or metastatic squamous cell carcinoma of the head and neck: 2-year long-term survival update of CheckMate 141 with analyses by tumor PD-L1 expression. Oral Oncol (2018) 81:45–51. doi: 10.1016/j.oraloncology.2018.04.008 29884413 PMC6563923

[54] Yarchoan M Hopkins A and Jaffee EM. Tumor mutational burden and response rate to PD-1 inhibition. N Engl J Med (2017) 377(25):2500–1. doi: 10.1056/NEJMc1713444 PMC6549688 29262275

[55] Goodman AM Kato S Bazhenova L Patel SP Frampton GM Miller V. Tumor mutational burden as an independent predictor of response to immunotherapy in diverse cancers. Mol Cancer Ther (2017) 16(11):2598–608. doi: 10.1158/1535-7163.Mct-17-0386 PMC5670009 28835386

[56] Moy JD Moskovitz JM Ferris RL. Biological mechanisms of immune escape and implications for immunotherapy in head and neck squamous cell carcinoma. Eur J Cancer (2017) 76:152–66. doi: 10.1016/j.ejca.2016.12.035 PMC5459368 28324750

[57] Chen SMY Krinsky AL Woolaver RA Wang X Chen Z and Wang JH. Tumor immune microenvironment in head and neck cancers. Mol Carcinog (2020) 59(7):766–74. doi: 10.1002/mc.23162 PMC7282929 32017286

[58] Seiwert TY Burtness B Mehra R Weiss J Berger R Eder JP. Safety and clinical activity of pembrolizumab for treatment of recurrent or metastatic squamous cell carcinoma of the head and neck (KEYNOTE-012): an open-label, multicentre, phase 1b trial. Lancet Oncol (2016) 17(7):956–65. doi: 10.1016/S1470-2045(16)30066-3 27247226

